# Perceptual restoration of locally time-reversed speech: Non-native listeners’ performance in their L2 vs. L1

**DOI:** 10.3758/s13414-021-02258-5

**Published:** 2021-04-16

**Authors:** Mako Ishida

**Affiliations:** Faculty of Science and Technology, Department of Foreign Language and Liberal Arts, Keio University, 4 Chome-1-1 Hiyoshi, Kohoku Ward, Yokohama, Kanagawa 223-8521 Japan

**Keywords:** Perceptual organization, Spoken word recognition, Psycholinguistics

## Abstract

Nonnative listeners are generally not as good as native listeners in perceptually restoring degraded speech and understand what was being said. The current study investigates how nonnative listeners of English (namely, native Japanese speakers who learned English as a second language) perceptually restore temporally distorted speech in their L2 English as compared with native English listeners (L1 English) reported in Ishida et al. (*Cognition, 151,* 68–75, 2016), and as compared with the listeners’ native tongue (L1 Japanese). In the experiment, listeners listened to locally time-reversed words and pseudowords in their L2 English and L1 Japanese where every 10, 30, 50, 70, 90, or 110 ms of speech signal was flipped in time—these stimuli contained either many fricatives or stops. The results suggested that the intelligibility of locally time-reversed words and pseudowords deteriorated as the length of reversed segments increased in both listeners’ L2 English and L1 Japanese, while listeners understood locally time-reversed speech more in their L1 Japanese. In addition, lexical context supported perceptual restoration in both listeners’ L1 Japanese and L2 English, while phonemic constituents affected perceptual restoration significantly only in listeners’ L1. On the other hand, locally time-reversed words and pseudowords in L1 Japanese were much more intelligible than those in L1 English reported in Ishida et al. It is possible that the intelligibility of temporally distorted lexical items depends on the structure of basic linguistic units in each language, and the Japanese language might have a unique characteristic because of its CV and V structure.

Perceptual restoration is a phenomenon where a person perceptually restores a missing or degraded signal as if it were intact. In fact, people perceptually restore acoustically distorted speech signal in daily situations and understand what was being said. For example, people can understand speech at a railroad station even if an announcement from a loudspeaker was masked by environmental noise. In addition, people can understand speech even when the announcement was interrupted by unexpected random sounds because of the malfunction of the loudspeaker. Further, people can understand speech even when the announcement was reverberated in a large hall. In general, people can perceptually restore the disrupted portion of speech, under certain conditions, by integrating multiple cues (Bashford, Reiner, & Warren, 1992; Cherry & Wiley, 1967; Houtgast, 1972; Kashino, 2006; Samuel, 1981a, 1981b; Warren, 1970; Warren, Obusek, & Ackroff, 1972). Presumably, people analyze the sound in their ear, transmit the acoustic information to their brain, mapping the sound information onto linguistic entities they know (e.g., phonemes, words, phrases, sentences), and understand speech (Liberman, Cooper, Shankweiler, & Studdert-Kennedy, 1967; Liberman & Mattingly, 1985; Marslen-Wilson & Tyler, 1980; McClelland & Elman, 1986; McQueen, Cutler, & Norris, 2006; Poeppel, 2003; Sjerps, Mitterer, & McQueen, 2011). In this process, language proficiency of listeners plays a significant role, and all sorts of phonemic, phonological, morphological, lexical, syntactic, sematic, and pragmatic knowledge work together to understand the unclear, distorted parts of speech (Bond, 1999; Voss, 1984;Warren, 1970; Warren & Obusek, 1971; Warren & Warren, 1970). In this regard, listeners’ language proficiency is largely involved in perceptual restoration.

Back in 1970, Richard Warren published two papers entitled “Perceptual restoration of missing speech sounds” and “Auditory illusions and confusions” and reported that people can understand speech even when a part of speech signal was deleted and replaced by extraneous sounds (Warren, 1970; Warren & Warren, 1970). In his experiments, he deleted a part of speech signal that corresponded to the sound of /s/ in “legislature” in a sentence, “The state governors met with their respective legislatures convening in the capital city,” and inserted the sound of a cough instead. Here, /s/ was deleted along with the transitional part of adjacent phonemes that can possibly provide perceptual cues to identify /s/. When participants listened to this sentence and were asked to identify which sound was deleted and replaced by the sound of the cough, nobody was able to identify the exact location of the cough. In fact, participants were seemingly unaware of the deleted sound of /s/. Rather, they perceptually restored the deleted /s/ and heard the illusory sound of it as if it were there. This auditory illusion was called “phonemic restoration” (later also “auditory induction” and “temporal induction”; Kashino & Warren, 1996; Warren, Bashford Jr., Healy, & Brubaker, 1994; Warren & Warren, 1970), and phonemic restoration was observed even if the extraneous sound was changed from the sound of a cough to a 1000-Hz tone (which intensity was the same as the peak intensity of the cough). On the other hand, when the deleted sound of /s/ was not replaced by other sounds and left as a complete silence, listeners were able to detect that /s/ was missing. It seems to suggest that a missing speech sound (i.e., the partial temporal distortion of speech signal) can be perceptually restored when there is an alternate sound covering the deleted part of speech.

In addition, Samuel (1981a, 1981b) reported that a deleted phoneme was perceptually restored when the deleted sound and replacing sound were acoustically similar. For example, a fricative consonant, which is produced by the air passing through the narrow constrictions in the vocal tract and represented with relatively aperiodic waveforms, was perceptually restored better when it was replaced by white noise than pure tone. On the other hand, a vowel, which is produced without any constrictions in the vocal tract and represented with relatively periodic waveforms, was perceptually restored better when it was replaced by pure tone than white noise. Overall, the amount of perceptual restoration was generally higher when the phonemes were replaced by white noise rather than pure tone. The acoustic similarity between the replaced and replacing sounds seems to be a key to successful perceptual restoration.

In fact, perceptual restoration follows the masking potential rules (Kashino, 2006; Warren & Sherman, 1974). That is, people can perceptually restore the deleted sound only when the replacing sound has enough spectral, temporal, and spatial characteristics to cover the gap in speech (Kashino, 2006; Samuel, 1981a, 1981b; Warren, 1970; Warren et al.,1972; Warren & Sherman, 1974; Warren & Warren, 1970). Warren and Warren (1970) reported that replacing sound needs to be as loud as or louder than the replaced sound. In addition, the center frequency of the replacing sound and replaced sounds need to be the same so that perceptual restoration takes place. In addition, as was described, the deleted sound and replacing sound need to be acoustically similar so that perceptual restoration takes place. Perceptual restoration does not happen when the gap of speech was remained as a silence. Perceptual restoration happens only when the gap of speech signal was covered by extraneous sounds that suffice above mentioned acoustic conditions. The restorability of the deleted speech signal is determined by the acoustic similarity between deleted sounds and replacing sounds.

In 1999, Saberi and Perrott reported another finding of perceptual restoration in their paper entitled “Cognitive restoration of reversed speech.” That is, people could understand speech even when the temporal sequence of speech was more drastically distorted (Saberi & Perrott, 1999). In their study, speech signals of spoken sentences were divided into equally timed segments from the onset of speech (e.g., 100 ms), and every segment was flipped in time (locally time-reversed speech). For example, when a 300-ms speech signal was divided into 100-ms segments from the onset of speech (i.e.,0–100, 100–200, 200–300), and when every segment was flipped in time, the temporal sequence of speech signal looked something like 100–0–200–100–300–200. While every segment of speech signal was played backward, people could understand speech by perceptually integrating the dispersed information in time.

Many follow-up studies supported this fact—people could understand spoken sentences as long as they were spoken at a normal speech rate and locally time-reversed (Greenberg & Arai, 2001; Ishida, Arai, & Kashino, 2018; Ishida, Samuel, & Arai, 2016; Kiss, Cristescu, Fink, & Wittmann, 2008; Magrin-Chagnolleau, Barkat, & Meunier, 2002; Remez et al., 2013; Stilp, Kiefte, Alexander, & Kluender, 2010; Ueda, Nakajima, Ellermeier, & Kattner, 2017). In general, people could understand spoken sentences almost perfectly when every 10–20 ms of speech signal was reversed in time. People could understand half of speech when every 60–70 ms of speech signal was reversed in time. People could hardly understand speech when every 90–100 ms of speech signal was reversed in time. This tendency was observed in many languages such as in English (Greenberg & Arai, 2001; Ishida et al., 2018; Remez et al., 2013; Stilp et al., 2010; Ueda et al., 2017), French (Magrin-Chagnolleau et al., 2002), German (Kiss et al., 2008; Ueda et al., 2017), Mandarin Chinese (Ueda et al., 2017), and Japanese (Nakajima, Matsuda, Ueda, & Remijn, 2018; Ueda et al., 2017). While previous studies of perceptual restoration suggested that a deleted speech signal was perceptually restored when acoustic conditions were met, the studies of locally time-reversed speech shed light on the fact that perceptual restoration takes place even when the acoustic constituents of speech were shifted from the original temporal position to the restorable range in time.

In fact, speech is intelligible when the temporal envelope of speech signal is relatively retained (Greenberg & Arai, 2001; Ishida et al., 2016; Ishida et al., 2018; Stevens, 1999). People can understand speech when every relatively short segment of speech is flipped in time, and this is because the temporal envelope of original speech is relatively preserved (Ishida et al., 2018). To recall, the manipulation of local time reversal alters the original speech signal into a new speech signal where no original temporal sequence of speech is available. Locally time-reversed speech does not follow any natural articulatory dynamics at all, as everything is played backward in every reversed segment. Within the reversed segment, there is no original formant transition. If the original formant transition was going from lower to higher frequency, for example, that would be represented from higher to lower in the reversed segment. In addition, there is no natural amplitude attenuation of speech from the onset of speech toward the end of articulation. The natural articulatory motion from the opening to closing of the mouth cannot be represented in locally time-reversed speech. Furthermore, the temporal sequence of phonemes such as CV (consonant + vowel), or the voice onset time (VOT) of stops, would be also represented in the “reversed” temporal order. In this regard, locally time-reversed speech contains a lot of unnatural articulatory artifacts. On the other hand, locally time-reversed speech also contains unnatural acoustic artifacts at the edge of reversed segments. For example, a speech signal of 0–100 ms and 100–200 ms will be represented as 100–0–200–100 ms in locally time-reversed speech, and the irrelevant parts of speech signal is artificially connected at the edge, which also generates additional clicks. In this regard, locally time-reversed speech contains a lot of acoustic artifacts that were not originally present. While locally time-reversed speech does not represent any natural articulatory movements at all and contains additional clicks, locally time-reversed speech is intelligible as long as the original temporal envelope of speech is relatively retained. This fact is also supported by earlier studies of locally time-reversed spoken sentences that discussed speech rate (Greenberg & Arai, 2001; Ishida, 2017; Remez et al., 2013; Stilp et al., 2010; Ueda et a., 2017). When speech rate is fast, for example, the articulatory movements (e.g., opening and closing of the mouth) are fast and overlapped in a short period of time, and the fast, overlapped articulatory gestures are represented in the temporal envelope of speech in a short period of time. Therefore, when local time reversal was imposed to fast speech where so much articulatory information is present in a short period of time, its intelligibility is also severely impaired. Locally time-reversed speech is intelligible as long as the original temporal envelope of speech is relatively retained—the temporal configuration of speech signal is a key to the robustness of speech.

While previous studies examined the intelligibility of locally time-reversed speech at a sentence level, Ishida et al. (2016) further examined the intelligibility of locally time-reversed speech at a lexical level, by limiting the available linguistic contexts to lexical and phonemic contexts (i.e., no sentential context was available). In this study, native English speakers listened to locally time-reversed English words and pseudowords that contained either many fricatives or stops, in order to see the effects of phonemic constituents on the intelligibility of locally time-reversed speech. These words and pseudowords were locally time-reversed at every 10, 30, 50, 70, 90, or 110 ms on a temporal axis. Their results suggested that the intelligibility of locally time-reversed words and pseudowords gradually dropped as the reversed segment length increased. In addition, native English speakers understood locally time-reversed words significantly better than locally time-reversed pseudowords. Also, fricative-dominant words and pseudowords were significantly more intelligible than stop-dominant words and pseudowords when locally time-reversed. In general, fricative consonants have relatively symmetric waveforms (with the air constantly flowing out of the mouth in the articulation), and stop consonants have relatively asymmetric waveforms (with a noise burst in the articulation by stopping the airflow and releasing the air). Therefore, the temporal envelope of fricative-dominant items tends to be better retained than stop-dominant items when locally time-reversed, and that seems to have supported the intelligibility of speech. Overall, the perceptual restoration of locally time-reversed speech at a lexical level was largely sustained by both lexical and acoustic-phonetic factors.

In addition, Kiss et al. (2008) added another perspective that perceptual restoration of locally time-reversed speech depends on listeners’ language proficiency. In their study, native German speakers and nonnative German speakers (who learned German for 2 to 18 years, and whose first language was either a Germanic, Romanic, and Slavic language) listened to locally time-reversed German sentences. These sentences were either semantically coherent, semantically incoherent, and pseudohomophonic sentences. Semantically coherent sentences were sentences that contained German words and made sense as a whole (i.e., sentential, lexical, and phonemic contexts were present). Semantically incoherent sentences were sentences that contained German words but did not make sense as a whole (i.e., lexical and phonemic contexts were present). Pseudohomophonic sentences were sentences that contained no German words but only German phonemes that lined up based on the German phonotactic rules (i.e., only phonemic context was present). When native and nonnative speakers listened to locally time-reversed speech of these sentences, they relatively understood both semantically coherent sentences (more than 90% and 80% intelligibility for native and nonnative speakers respectively when every 38 ms of speech was reversed) and semantically incoherent sentences (more than 90% and 60% intelligibility for native and nonnative speakers respectively when every 38 ms of speech was reversed), but they hardly understood pseudohomophonic sentences (around 10% and 0% intelligibility for native and nonnative speakers, respectively, when every 38 ms of speech was reversed). Here, both native and nonnative speakers understood semantically coherent sentences better than semantically incoherent sentences, although native speakers understood locally time-reversed speech better than nonnative speakers did. While semantic contexts strongly supported both native and nonnative speakers’ perceptual restoration, native speakers performed better than nonnative speakers, and language proficiency strongly affected perceptual restoration. The more familiar the listeners are with the target language and the more contexts are available for listeners, the more successful perceptual restoration can be expected.

In fact, language proficiency was also described as one of the key factors for perceptual restoration in earlier studies. Warren and Warren (1970) reported, by citing an unpublished study by Gary Sherman in their lab, that a deleted phoneme was perceptually restored based on sentential contexts. For example, when a word “(?)eel” was placed in a sentence “It was found that the (?)eel was on the __”, the deleted phoneme “(?)” was perceptually restored depending on the last word of the sentence “__.” When the last word was “axle,” “shoe,” “orange,” and “table,” a word with the deleted phoneme “(?)” was understood as “wheel,” “heel,” “peel,” and “meal,” respectively. Listeners seem to retain ambiguous parts of speech until they come across necessary information to understand speech. Moreover, Warren and Sherman (1974) reported that listeners did not perceptually restore the missing speech sound as was originally pronounced. Rather, they relied on the sentential context to restore the deleted phonemes. For example, when a pseudoword “sandwagon” was pronounced in a sentence, “It is common for people to jump on the sandwagon when a political movement becomes popular,” and when the first /s/ in “sandwagon” was deleted and replaced by the sound of a cough, people perceptually restored the deleted phoneme as /b/ and understood the word as “bandwagon.” While the acoustic factors are important for perceptual restoration at a phoneme level, the contextual information also plays a dominant role in perceptual restoration. People can understand the ambiguous part of speech based on the linguistic context.

Moreover, listeners’ familiarity to the target sound itself also affects perceptual restoration of ambiguous speech sound. McQueen et al. (2006) suggested that people categorize ambiguous speech sound based on their experience. When a person was exposed to an ambiguous speech sound that is in the continuum between [f] and [s], and when the person learned that the ambiguous sound was [f] in the training session, the person tended to be biased to judge other similar ambiguous speech sounds as [f] even after the training session. Also, if a person learned that the ambiguous sound was [s], the person tended to be biased to judge other similar ambiguous sounds as [s]. People seem to process acoustically similar sounds based on their prior knowledge. Additionally, Sjerps et al. (2011) suggested that people perceived the ambiguous vowel sound based on the acoustic characteristics of its preceding sound. When the preceding speech sound was high in its first formant frequency (F1), the following ambiguous vowel between [ɪ] and [ɛ] was perceived as [ɪ]. On the other hand, when the preceding speech sound was low in its F1, the following ambiguous vowel was perceived as [ɛ]. Listeners understood ambiguous vowel sounds based on their prior experience. In fact, people process various speech sounds in daily life, and people can understand speech even when different talkers pronounced the target sound in different ways. The exposure to the target speech sound as well as listeners’ prior experience seem to support perceptual restoration of ambiguous speech sounds.

The current study further investigates how people understand temporally disrupted speech at a lexical level in their second language which listeners presumably have less exposure to and knowledge about. Specifically, this study examines how native Japanese speakers who learned English as a second language perceptually restore locally time-reversed words and pseudowords in their L2 English as compared with native English speakers (L1 English) reported in Ishida et al. (2016), and as compared with the listeners’ own native tongue (L1 Japanese), by adopting procedures from Ishida et al. (2016). There are four independent variables in this study: language (English vs. Japanese), reversed segment length (10, 30, 50, 70, 90, and 110 ms), lexicality (words vs. pseudowords), and dominant consonants in the stimuli (fricative-dominant vs. stop-dominant). While it is predictable and not surprising that people do not perform well in their second language (L2) as compared with their first language (L1) and as compared with the native speakers of the target language, the current study examines what specifically is different between listeners’ performance in their first and second language.

## Method

### Participants

A total of 60 native Japanese speakers who spoke English as a second language (48 females, 12 males) were recruited at Sophia University in Tokyo, Japan. Their average age was 23 years old. Their English proficiency was intermediate, based on the placement test of DIALANG (Alderson, 2006; Lancaster University, 2014), which measured the vocabulary size of test-takers. The average score of the placement test was 483 out of 1,000 full marks, the third level of proficiency from the top, out of six levels. The participants did not report any hearing or speech impairments. The participants received monetary remuneration for their participation.

### The English materials

A set of 60 fricative-dominant words and 60 fricative-dominant pseudowords, and a set of 60 stop-dominant words and 60 stop-dominant pseudowords were selected from the materials of Ishida et al. (2016) (see Appendix A). All words were originally selected from MRC Psycholinguistic Database (1997), and were three, four, or five syllables long. The selected words had a Kučera and Francis (1967) written frequency greater than 1/million, and a familiarity rating of 500–700 in the MRC database. The mean frequency of fricative-dominant words was 19.32, and the mean frequency of stop-dominant words was 21.24 in the CLEARPOND database (Marian, Bartolotti, Chabal, & Shook, 2012). Fricative-dominant words contained relatively more fricatives (*M* = 1.70) than stops (*M* = 0.75), while stop-dominant words contained relatively more stops (*M* = 3.22) than fricatives (*M* = 0.53), in which *M* denotes the average number of the target phonemes (fricative or stop) in the selected words. Here, working within the constraints of phonemic occurrence in English, the number of fricatives in fricative-dominant words was almost 3.2 times greater than that in stop-dominant words, while the number of stops in stop-dominant words was almost 4.3 times greater than that in fricative-dominant words. Matched pseudowords were created by replacing a phoneme in a word with another phoneme that had a different place of articulation (e.g., “academic” with [d] vs. “acabemic” with [b]). The target phoneme for manipulation was the first consonant of either the second, third, or fourth syllable. All items were spoken by one male and one female native American English speakers and recorded in a sound proof room by using a digital audio recorder (Tascam HD-P2) and a microphone (Crown CM-311A). The speech was first digitized at 48 kHz (16 bit), and then down-sampled to 16 kHz (16 bit) and stored as WAV files. The amplitude of speech signal was normalized based on the peak level by using the GoldWave digital audio editing software. The speech signals of words and pseudowords in the male voice were locally time-reversed at every 10, 30, 50, 70, 90, or 110 ms. The joints of the adjacent reversed segments were cross-faded, and the amplitude envelope was linearly adjusted with a tapering length of 5 ms, in order to reduce possible additional noise and clicks. The manipulation of speech signals was conducted by using MATLAB.

### The Japanese Materials

A set of 60 fricative-dominant words and 60 stop-dominant words were selected from “Nihongo no goi tokusei” (Amano & Kondo, 2000), a database of Japanese words (see Appendix B). This database contained Japanese words with “presentation-dependent” measures of familiarity: “orthography + sound” familiarity, sound-alone familiarity, and orthography-alone familiarity. The familiarity was rated from 1 to 7 (1 = *unfamiliar*, 7 = *familiar*) for each word in the database. The average familiarity rating of selected fricative-dominant words was 5.61, and that of selected stop-dominant words was 5.66. All words were 4 morae long (CVCVCVCV), and they had no special mora of the Japanese language. After selecting the fricative-dominant and stop-dominant words, matched pseudowords were created by replacing a target phoneme with another phoneme that had a different place of articulation (e.g., “kakutoku” with [k] was changed to “kaputoku” with [p]). The target phoneme was always the first phoneme of either the second, third, or fourth mora. Fricative-dominant words contained relatively many fricatives (*M* = 2.13) as compared with stops (*M* = 1.02), while stop-dominant words contained relatively many stops (*M* = 3.25) as compared with fricatives (*M* = 0.17). Working within the constraints of phonemic occurrence in Japanese, the number of fricatives in fricative-dominant words was almost 12.5 times greater than that in stop-dominant words, while the number of stops in stop-dominant words was almost 3.2 times greater than that in fricative-dominant words. The selected words were spoken by one male and one female native Japanese speakers and recorded in a soundproof room by using a digital audio recorder (SONY PCM-D50) and a microphone (SONY ECM-MS957). The speech was recorded at a sampling rate of 44.1 kHz with 16-bit resolution, and saved as WAV files. The amplitude of speech signal was normalized based on the peak level by using the GoldWave digital audio editing software. Words and pseudowords in the male voice were locally time-reversed at every 10, 30, 50, 70, 90, or 110 ms by MATLAB. The joint parts of adjacent reversed segments were cross-faded and a linear amplitude adjustment was imposed with a tapering length of 5 ms to reduce possible additional clicks or noise.

### Procedure

The experiment procedure was adopted from Ishida et al. (2016), and there were four independent variables in this study: language (English vs. Japanese), reversed segment length (10, 30, 50, 70, 90, and 110 ms), lexicality (words vs. pseudowords), and dominant consonants in the stimuli (fricative-dominant vs. stop-dominant). Participants were first randomly assigned to either fricative-dominant stimuli group or stop-dominant stimuli group. Then, in each group, participants experienced two sessions (i.e., English session and Japanese session) where participants listened to locally time-reversed words and pseudowords in English and in Japanese. For counterbalancing purposes, half of the participants started with the English session, followed by the Japanese session, and the other half started with the Japanese session, followed by the English session.

The experiment was conducted in a sound-shield room in Sophia University, Tokyo, Japan. Participants listened to the stimuli in front of a computer monitor wearing headphones (SONY MDR-CD900ST). The stimuli were presented diotically at a participant’s comfortable listening level throughout each participant’s experiment session. A USB audio interface (Roland UA-25 EX) was placed between the computer’s USB port and the headphones as a digital to analog converter. Participants listened to a pair of lexical items in each trial. The first lexical item was spoken by a male speaker and locally time-reversed, and the second lexical item was spoken by a female speaker and it was not locally time-reversed, as was originally designed in Ishida et al. (2016). Participants were instructed in Japanese to listen to a pair of words and judge whether the first and second items were the same or different. The Japanese instructions were given as, “You are going to listen to a pair of words in which the first word was acoustically distorted and the second word was normally spoken. Please judge whether the first and second lexical items were the same or different words, by using the same or different button on your response pad.” There were four kinds of pairs: word–word (same), pseudoword–pseudoword (same), word–pseudoword (different), and pseudoword–word (different). The gender of the speaker for the first and second lexical item was intentionally changed so that listeners judged “same” or “different” not based on the similarity of the sound quality but based on the lexical items they had in mind, as was originally designed in Ishida et al. (2016). In each trial, listeners responded “same” or “different” by pressing a button on a response pad. Each participant experienced 240 trials in each session, with 4 pairs × 60 items in a randomized order. Within the four pairs, two pairs were with a locally time-reversed word (word–word for “same,” word–pseudoword for “different”), and the other two pairs were with a locally time-reversed pseudoword (pseudoword–pseudoword for “same,” pseudoword–word for “different”). The 60 items were composed of six subsets of 10 items, with each subset assigned to one of the six segment durations (10, 30, 50, 70, 90, and 110 ms). The subset was the subdivision of a set of 60 fricative-dominant words, 60 fricative-dominant pseudowords, 60 stop-dominant words, and 60 stop-dominant pseudowords. Six groups of participants, in a Latin square, were used to counterbalance the six subsets of items across the six segment durations for each session. The interstimulus interval was 400 ms, and the total duration of the experiment with two sessions was approximately 40 minutes.

## Results

To recall, the current study adopted the experiment paradigm of Ishida et al. (2016), and examined the intelligibility of locally time-reversed words and pseudowords in listeners’ L2 English versus L1 Japanese by adopting the same/different task. Listeners judged whether the first presented locally time-reversed lexical item and the second presented nonreversed lexical item were “same” or “different.” Words and pseudowords adopted here were different only by one phoneme, and this subtle difference was intentionally created in order to see how sensitive participants were in perception of locally time-reversed words and pseudowords. For analysis, the *d'* of the signal detection theory (along with the response bias, β) was computed for each subject, for their responses to locally time-reversed words and pseudowords in which every 10, 30, 50, 70, 90, and 110 ms of speech signal was flipped in time. The *d'* value was adopted as an indicator of the intelligibility of locally time-reversed words and pseudowords in both listeners’ L2 English and L1 Japanese. Miss rates and false-alarm rates were calculated for error responses where participants responded either “same” when the paired words were different or “different” when the paired words were the same. When a miss rate or false alarm rate was at ceiling or floor, it was replaced by the value of 1/2*N* (floor) or 1 − 1/2*N* (ceiling) where *N* denotes the number of items.

**Fig. 1:**
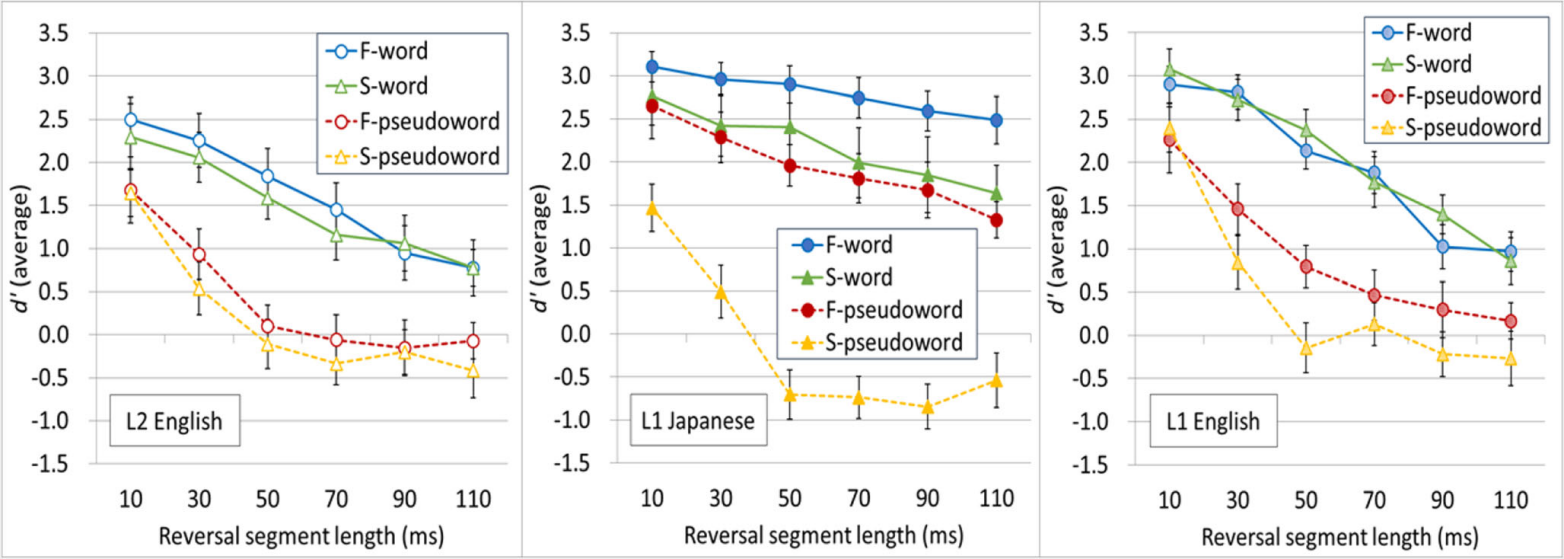
Intelligibility of locally time-reversed fricative-dominant and stop-dominant words and pseudowords in *d'*. The left panel shows the intelligibility of locally time-reversed lexical items in listeners’ L2 English, the middle panel shows the intelligibility in listeners’ L1 Japanese, and the right panel shows the intelligibility in listeners’ L1 English reported in Ishida et al. (2016) for comparison. (Color figure online)

Figure 1 shows the intelligibility of locally time-reversed words and pseudowords measured in *d'*. The left panel shows the intelligibility of locally time-reversed words and pseudowords in listeners’ L2 English, the middle panel shows the intelligibility of locally time-reversed words and pseudowords in listeners’ L1 Japanese, and the right panel shows the intelligibility of locally time-reversed words and pseudowords in listeners’ L1 English reported in Ishida et al. (2016). As expected, native Japanese speakers who learned English as a second language did not perform well in their L2 English as compared with native English speakers (L1 English), and as compared with their own mother tongue (L1 Japanese). As a general tendency, words were more intelligible than pseudowords, and fricative-dominant stimuli were more intelligible than stop-dominant stimuli when locally time-reversed. In addition, the intelligibility of locally time-reversed items gradually deteriorated when the reversed segment length increased. Also, the intelligibility of stop-dominant pseudowords dropped drastically after the reversed segment length was extended from 10 to 30 ms. In addition, locally time-reversed words and pseudowords in L1 Japanese (middle panel) were, generally, perceptually restored better than locally time-reversed words and pseudowords in L1 English (right panel).

An analysis of variance (ANOVA) was performed for the results in listeners’ L2 English and L1 Japanese respectively, with phoneme type (fricative-dominant vs. stop-dominant stimuli) as a between-subjects factor, and with lexical status (word vs. pseudoword), and reversed segment length (10, 30, 50, 70, 90, 110 ms) as within-subjects factors. An ANOVA for the results in listeners’ L2 English suggested that the intelligibility of locally time-reversed items deteriorated significantly when the reversed segment length increased, *F*(5, 290) = 146.33, *p* < .001, partial η^2^ = .72. In addition, words were significantly more intelligible than pseudowords, *F*(1, 58) = 140.28, *p* < .001, partial η^2^ = .71. However, fricative-dominant lexical items were *not* significantly different from stop-dominant lexical items, *F*(1, 58) = 2.87, *p* = .096, partial η^2^ = .05. There was a significant interaction between lexical status and reversed segment length, *F*(5, 290) = 10.35, *p* < .001, partial η^2^ = .15, which seems to suggest that lexical contexts greatly supported perceptual restoration by nonnative listeners (word > pseudoword). On the other hand, there was no significant interaction between lexical status and phoneme type, *F*(1, 58) = .13, *p* = .72, partial η^2^ = .00, and no significant interaction between the reversed segment length and phoneme type, *F*(5, 290) = 1.20, *p* = .31, partial η^2^ = .02, and no significant three-way interaction among lexical status, reversed segment length, and phoneme type, *F*(5, 290) = .73, *p* = .6, partial η^2^ = .01, which seems to suggest that the intelligibility gap between words and pseudowords in the fricative-dominant stimuli group was not significantly different from that in the stop-dominant stimuli group. The difference in phoneme type (fricative vs. stop) did not affect the intelligibility of lexical items (words vs. pseudowords) along with the extension of reversed segment length. The above results suggested that the way nonnative listeners perceptually restored locally time-reversed words and pseudowords in their L2 English was both similar to and different from the way native listeners did in their L1 English in Ishida et al. (2016). One of the similarities was that the intelligibility of locally time-reversed speech deteriorated significantly when the reversed segment length increased. Both native and nonnative listeners understood locally time-reversed words well when the reversed segment length was around 10–30 ms, but the intelligibility of locally time-reversed words dropped when the reversed segment length was around 70–90 ms. Another similarity was that locally time-reversed words were significantly more intelligible than locally time-reversed pseudowords, and lexical context supported perceptual restoration greatly. On the other hand, in nonnative listeners’ perceptual restoration, the acoustic-phonetic properties did not affect the perceptual restoration processes as much as they did for native listeners. That is, fricative-dominant items were not significantly more intelligible than stop-dominant items. Overall, nonnative listeners’ perceptual restoration in this study was more influenced by lexical factors than acoustic-phonetic factors.

Another ANOVA for the results in listeners’ L1 Japanese, with Huynh–Feldt correction, also suggested that the intelligibility of locally time-reversed lexical items deteriorated significantly when the reversed segment length was extended, *F*(4.6, 266.67) = 98.07, *p* < .001, partial η^2^ = .63, which was also observed in listeners’ second language. In addition, words were significantly more intelligible than pseudowords, *F*(1, 58) = 158.06, *p* < .001, partial η^2^ = .73, which was also observed in listeners’ second language. On the other hand, fricative-dominant items were significantly more intelligible than stop-dominant items, *F*(1, 58) = 116.39, *p* < .001, partial η^2^ = .67, which was not observed in listeners’ L2 English. Overall, there was a significant interaction between reversed segment lengths and lexical status, *F*-(4.55, 263.64) = 16.02, *p* < .001, partial η^2^ = .22, which shows lexical advantages (word > pseudoword) in perceptual restoration. In addition, there were significant interactions between lexical status and phoneme type, *F*(1, 58) = 34.21, *p* < .001, partial η^2^ = .37, and between reversed segment length and phoneme type, *F*(4.6, 266.66) = 12.39, *p* < .001, partial η^2^ = .18, which seems to suggest the influence of acoustic-phonetic factors (fricative > stop) in perceptual restoration. There was also a significant three-way interaction among reversed segment length, lexical status, and phoneme type, *F*(4.55, 263.64) = 5.93, *p* < .001, partial η^2^ = .09, which shows the intelligibility gap between words and pseudowords in fricative-dominant stimuli group along with the extension of reversed segment length, was significantly different from that in stop-dominant stimuli group.

Overall, the intelligibility of locally time-reversed lexical items gradually deteriorated significantly when the reversed segment length was extended, in both listeners’ L2 English and L1 Japanese. In addition, listeners understood locally time-reversed words significantly better than pseudowords in both listeners’ L2 English and L1 Japanese. On the other hand, listeners understood fricative-dominant items significantly better than stop-dominant items in their L1 Japanese (which was also observed with L1 English listeners reported in Ishida et al., 2016), while the significant difference was not observed in their L2 English. It seems that lexical context strongly supports perceptual restoration of locally time-reversed lexical items in both listeners’ first and second language, but acoustic-phonetic properties affect perceptual restoration of locally time-reversed lexical items only in listeners’ first language (or, possibly, only when nonnative listeners are very proficient in the target language).

**Fig. 2:**
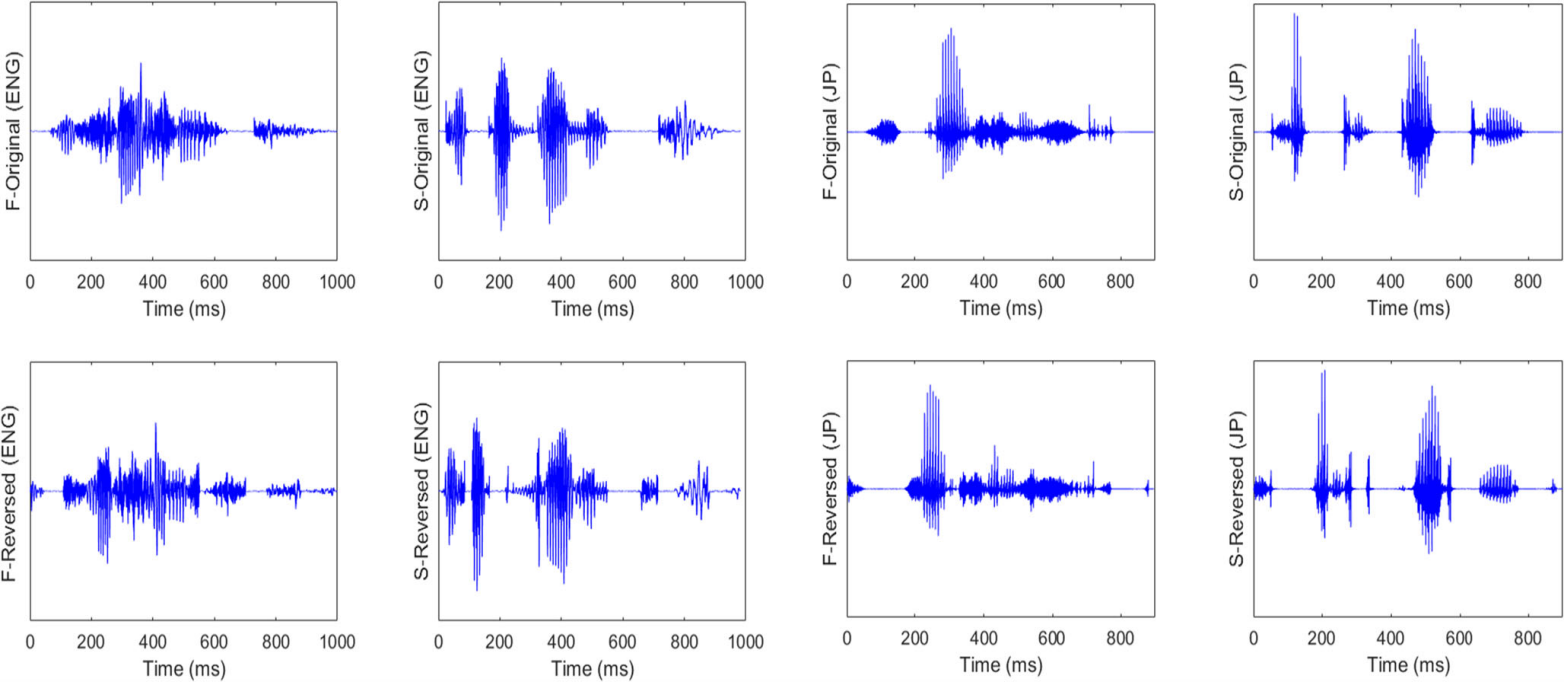
Waveforms of fricative-dominant words (F-) versus stop-dominant words (S-). The upper panel shows the waveforms of naturally spoken words (F-original, S-original) in English (ENG) and Japanese (JP). The lower panel shows the waveforms of locally time-reversed words (F-reversed, S-reversed) in English (ENG) and Japanese (JP) where every 110 ms of speech signal was flipped in time. Here, fricative-dominant and stop-dominant words in English were “associate” and “academic” respectively, and those in Japanese were “shitashisa” (= intimacy) and “kakutoku” (= acquire) respectively.

In order to look at how much acoustic-phonetic properties affected perceptual restoration, and how similarly or differently the same individuals performed when confronted with the temporal distortion in their first and second language, an additional ANOVA was performed with phoneme type (fricative-dominant vs. stop-dominant stimuli group) as a between-subjects factor, and language (English vs. Japanese), lexical status (word vs. pseudoword), and reversed segment length (10, 30, 50, 70, 90, and 110 ms) as within-subject factors, along with Huynh–Feldt correction. The results suggested that fricative-dominant stimuli were significantly more intelligible than stop-dominant stimuli, *F*(1, 58) = 62.12, *p* < .001, partial η^2^ = .52. In addition, listeners understood locally time-reversed speech significantly better in their first language (Japanese) than in their second language (English), *F*(1, 58) = 149.77, *p* < .001, partial η^2^ = .72. Also, words were significantly more intelligible than pseudowords, *F*(1, 58) = 217.21, *p* < .001, partial η^2^ = .79. At the same time, the intelligibility of locally time-reversed speech deteriorated significantly when the reversed segment length was extended, *F*(4.72, 273.63) = 226.68, *p* < .001, partial η^2^ = .80. There was a significant interaction between language and phoneme type, *F*(1, 58) = 88.02, *p* < .001, partial η^2^ = .60, which suggests that the intelligibility gap between fricative-dominant and stop-dominant stimuli in listeners’ first language was significantly different from that in listeners’ second language. In fact, acoustic-phonetic factors significantly influenced the intelligibility of locally time-reversed lexical items in listeners’ first language, but not in listeners’ second language. There was also a significant interaction between language and lexical status, *F*(1, 58) = 6.10, *p* = .016, partial η^2^ = .10, which suggests that the intelligibility gap between locally time-reversed words and pseudowords in listeners’ first language was significantly different from that in listeners’ second language. Lexical contexts supported perceptual restoration in listeners’ first language more than in listeners’ second language. Also, there was a significant interaction between phoneme type and lexical status, *F*(1, 58) = 16.13, *p* < .001, partial η^2^ = .22, which suggests that the intelligibility gap between words and pseudowords in fricative-dominant stimuli was significantly different from that in stop-dominant stimuli. In fact, fricative-dominant words and pseudowords retained their intelligibility relatively higher than stop-dominant words and pseudowords when locally time-reversed. The acoustic structure of fricatives (i.e., relatively symmetric waveforms) seems to have provided advantages in perceptual restoration of locally time-reversed speech as compared with stops (i.e., relatively asymmetric waveforms; see Fig. 2). In addition, there were significant interactions between reversed segment length and language, *F*(4.70, 272.33) = 7.56, *p* < .001, partial η^2^ = .12, between reversed segment length and phoneme type, *F*(4.72, 273.63) = 5.60, *p* < .001, partial η^2^ = .09, and between reversed segment length and lexical status, *F*(4.01, 232.30) = 20.69, *p* < .001, partial η^2^ = .26, which suggests that the way the intelligibility of locally time-reversed lexical items deteriorated along with the extension of reversed segment length was significantly different between listeners’ first and second language, between fricative-dominant and stop-dominant stimuli, and between words and pseudowords, respectively. Furthermore, there was a significant three-way interaction among lexical status, phoneme type, and language, *F*(1, 58) = 28.47, *p* < .001, partial η^2^ = .33, which suggests that the intelligibility gap between words and pseudowords was significantly different between fricative-dominant and stop-dominant stimuli, and this gap was also significantly different between L1 Japanese and L2 English. Also, there was a significant interaction among reversed segment length, phoneme type, and language, *F*(4.70, 272.33) = 6.51, *p* < .001, partial η^2^ = .10, which suggests that the way intelligibility deteriorated along with the extension of reversed segment length in fricative-dominant stimuli was significantly different from that in stop-dominant stimuli, and this gap was also significantly different between L1 Japanese and L2 English. There were also a significant interaction among reversed segment length, lexical status, phoneme type, *F*(4.01, 232.30) = 2.65, *p* = .034, partial η^2^ = .04, and a significant interaction among reversed segment length, lexical status, and language, *F*(5, 290) = 4.01, *p* = .002, partial η^2^ = .07, which suggests that the intelligibility gap between words and pseudowords along with the extension of reversed segment length was significantly different between fricative-dominant and stop-dominant stimuli, and between L1 Japanese and L2 English respectively. Overall, a significant four-way interaction was observed among reversed segment length, lexical status, language, and phoneme type, *F*(5, 290) = 3.87, *p* = .002, partial η^2^ = .06, which suggests that the intelligibility gap between words and pseudowords, along with the extension of reversed segment length, was different between fricative-dominant stimuli and stop-dominant stimuli, and this overall phenomenon was significantly different between listeners’ L1 Japanese and L2 English. On the other hand, it should be also noted here that these statistical differences might be also attributed to stop-dominant stimuli (especially, pseudowords) that tended to lose their intelligibility drastically when the reversed segment length exceeded 30 ms. Stop-dominant stimuli were generally more susceptible to the local time reversal and tended to lose their intelligibility earlier than fricative-dominant stimuli, and this might have been one of the factors that generated the statistical difference in the pooled analysis here. However, it is also interesting to note that stop-dominant “words” retained their intelligibility overall when the reversed segment length was extended, while stop-dominant “pseudowords” lost their intelligibility drastically already when the reversed segment length was increased from 30 ms to 50 ms. The intelligibility gap between stop-dominant words and pseudowords might well reflect lexical advantage in perceptual restoration.

**Fig. 3:**
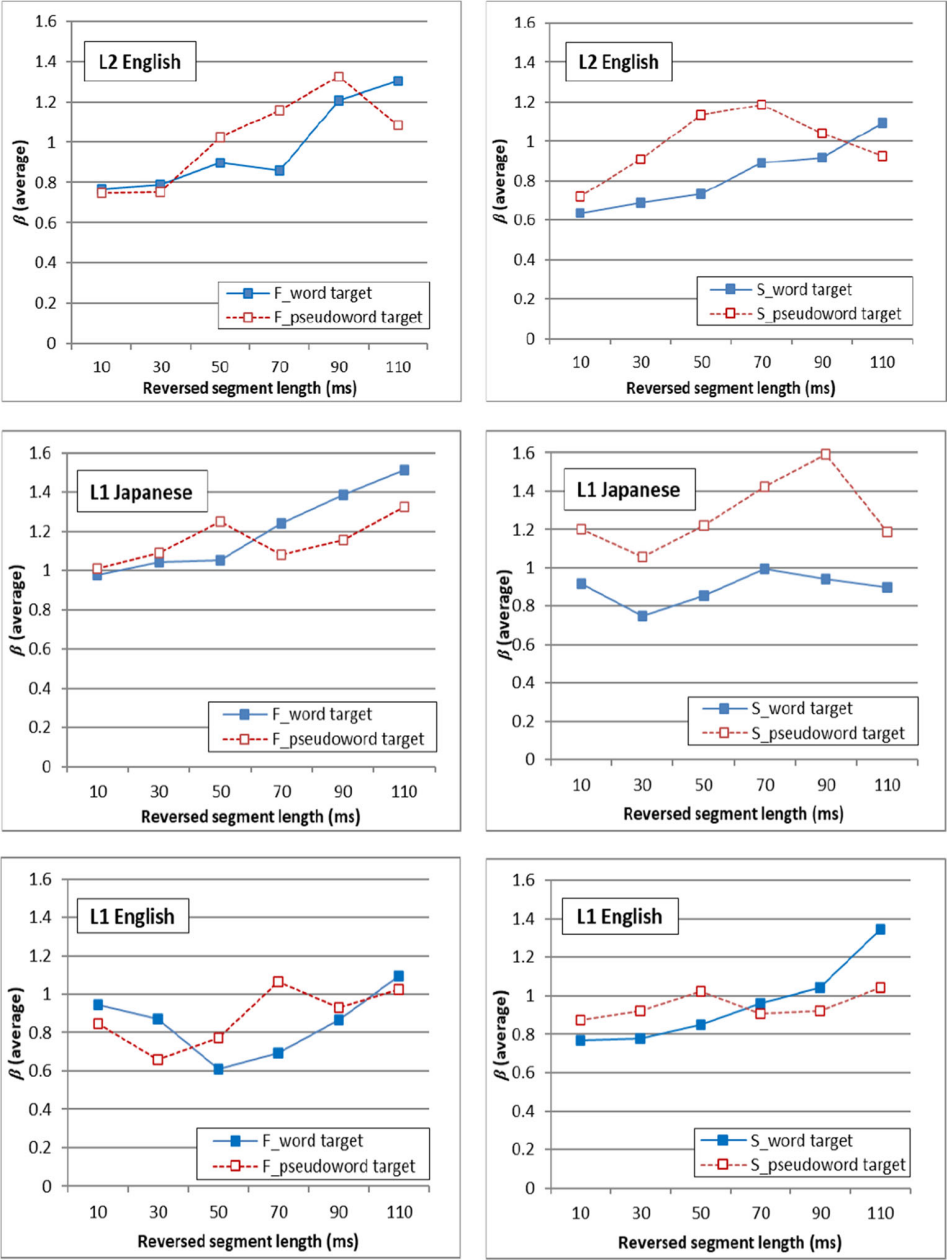
Response bias represented in β of the signal detection theory. The left panels show the response bias for locally time-reversed fricative-dominant stimuli in listeners’ L2 English and L1 Japanese as well as L1 English which data are coming from Ishida et al. (2016), and the right panels show the response bias for locally time-reversed stop-dominant stimuli. Here, β = 1 means neutral, β > 1 means listeners were biased to respond “different,” and β < 1 means listeners were biased to respond “same” in the current study design.

The analysis of *d'* of the signal detection theory also made it possible to compute the response bias, β*.* Figure 3 shows the response bias represented in β. In the current study design, β > 1 means that the participants tended to respond “different,” and β < 1 means that the participants tended to respond “same,” while β = 1 means that the listeners’ response was not biased and neutral. The average β for the reversed segment length of 10, 30, 50, 70, 90, and 110 ms was 0.77, 0.79, 0.90, 0.86, 1.21, and 1.30, respectively, for fricative-dominant words in L2 English, 0.75, 0.76, 1.02, 1.16, 1.32, and 1.09 for fricative-dominant pseudowords in L2 English, 0.64, 0.69, 0.73, 0.89, 0.92, and 1.09 for stop-dominant words in L2 English, and 0.72, 0.91, 1.13, 1.18, 1.04, and 0.93 for stop-dominant pseudowords in L2 English. In addition, the average β for the reversed segment length of 10, 30, 50, 70, 90, and 110 ms was 0.98, 1.04, 1.05, 1.24, 1.38, 1.51, and 1.20 for fricative-dominant words in L1 Japanese, 1.01, 1.09, 1.25, 1.08, 1.15, and 1.32 for fricative-dominant pseudowords in L1 Japanese, 0.92, 0.75, 0.86, 1.00, 0.94, and 0.90 for stop-dominant words in L1 Japanese, and 1.20, 1.06, 1.22, 1.42, 1.59, and 1.19 for stop-dominant pseudowords in L1 Japanese. Also, an additional analysis of the average β obtained in Ishida et al. (2016) suggested the average β for the reversed segment length of 10, 30, 50, 70, 90, and 110 ms was 0.94, 0.87, 0.61, 0.69, 0.87, and 1.09 for fricative-dominant words in L1 English, 0.85, 0.66, 0.77, 1.06, 0.93, and 1.02 for fricative-dominant pseudowords in L1 English, 0.77, 0.78, 0.85, 0.96, 1.04, and 1.35 for stop-dominant words in L1 English, and 0.87, 0.92, 1.02, 0.91, 0.92, and 1.04 for stop-dominant pseudowords in L1 English. As the graph shows, there is no consistent common trend across different languages, lexical types, and phoneme type. However, the results of stop-dominant stimuli in L1 Japanese showed that people tended to be a little biased to say “different” for pseudoword stimuli, and “same” for word stimuli. There was also a very weak trend overall that the β score went up as the reversed segment length increased, but this trend was not consistent. The analysis of β score of the signal detection theory did not suggest any consistent trends overall.

In sum, native Japanese speakers who learned English as a second language performed poorer in their second language (L2 English) as compared with native English speakers (L1 English) and as compared with their own mother tongue (L1 Japanese). Also, words were more intelligible than pseudowords when locally time-reversed in both L2 English and L1 Japanese, as was also observed in L1 English in Ishida et al. (2016). On the other hand, fricative-dominant lexical items were significantly more intelligible than stop-dominant lexical items in L1 Japanese and L1 English, but not in L2 English. Listeners in the current study perceptually restored locally time-reversed words and pseudowords better in their L1 than in their L2, and lexical advantage permeated in perceptual restoration of locally time-reversed lexical items, while acoustic-phonetic factors influenced only perceptual restoration processes in listeners’ L1 and not in their L2.

## Discussion

The current study explored how native Japanese speakers who learned English as a second language perceptually restore locally time-reversed words and pseudowords (that were either fricative-dominant or stop-dominant in their phonemic constituents) in their L2 English and L1 Japanese as compared with L1 English reported in Ishida et al. (2018). The results suggested that there was lexical advantage in perceptual restoration of locally time-reversed lexical items in listeners’ L2 English and L1 Japanese as well as L1 English, but acoustic-phonetic properties only affected perceptual restoration in listeners’ L1 Japanese and L1 English, but not in L2 English. Lexical advantage was observed in perceptual restoration of locally time-reversed items in listeners’ first and second language, but phonemic constituents of speech affected perceptual restoration in listeners’ first language only. In other words, lexical advantage may be used to a similar extent by both native and nonnative listeners, but acoustic-phonetic information may be used by native listeners only, or when listeners are proficient in the target language.

As for lexical advantage in perceptual restoration, native Japanese speakers who learned English as a second language, perceptually restored locally time-reversed words significantly better than pseudowords in both L1 Japanese and L2 English. Here, lexical advantage permeated in perceptual restoration processes. To recall, the English proficiency of the participants in this study was intermediate based on DIALANG, which measured language proficiency based on the approximate vocabulary size (Alderson, 2006; Lancaster University, 2014), and English words used for this study were high-frequency words. So, the listeners in this study possibly enjoyed lexical advantage in perceptual restoration also in their L2 English as the target words were relatively familiar to them. In fact, Nation (2001) suggested that the vocabulary size largely affects the understanding of the target language. While the vocabulary size of well-educated native English speakers was estimated to be around 20,000 word-families (Goulden, Nation, & Read, 1990; Nation, 2001; Zechmeister, Chronis, Cull, D’Anna, & Healy, 1995), the ideal vocabulary size of nonnative English speakers for reading was estimated to be around 8,000-9,000 word-family vocabulary and that for listening was 6,000-7,000 word-family vocabulary (Nation, 2006). Hirsh and Nation (1992) also suggested, from their study about reading, second language learners need to know around 98%–99% of words in texts in order to understand and enjoy the texts, which means there is only one unknown word in every 50–100 words. When it comes to listening in adverse conditions in the second language, second language learners need to familiarize themselves to vocabulary in the first place to enjoy lexical support in perceptual restoration, but listeners also need to know a variety of sounds in association with target words.

In fact, people cope with varying speech sounds in daily situations. Johnson (2004) analyzed the corpus of American English called “Variation in Conversation Corpus” (38,560 content words, 49,362 function words; Pitt, Johnson, Hume, Kiesling, & Raymond, 2003) and suggested that people tend to deviate (delete or change) phonemes when they pronounce words in daily conversations. For example, a word “until” [∧ntɪl] was pronounced as [ɛntɪl] with a phoneme [ɛ], or [ənt∧_] with phonemes [ə] and [∧] along with one phoneme omitted, or [__tə_] with [ə] and three phonemes missing. While people perceptually restore the deviated portion of speech in daily situations, there seems to be a permissible range of deviation from citation forms for people to understand speech—overall, people could perceptually restore up to 20% deletion and 40% deviation (deletion + change) of phonemes for function words, and 10% deletion and 25% deviation for content words. Looking at these data, there seems to be a more variety of pronunciation for function words, but content words also entail drastic change in their pronunciation. For example, Dalby (1986) reported that people tend to speak fast in daily situations, and a word “probably” [prɑbəbli] was pronounced as [prɑbli], [prɑli], and [prɑ] by deleting consonants and vowels. Also, a word “seven” [sɛvn] was pronounced as [sɛm] by replacing and deleting consonants. Johnson (2004) also reported that a word “hilarious” [hɪlɛri∧s] was pronounced as [hlɛrɛs] by deleting and deviating vowels, and a word “particular” [p^h^ət^h^ɪk^h^jəl] was pronounced as [p^h^t^h^ɪk^h^] by deleting vowels and consonants. Additionally, Brown and Hilferty (1982, 1995) reported that “Did you eat yet?” in casual speech was pronounced something like “J’eat jet?,” and “Where are you?” was pronounced something like “Wheraya?.” When people speak fast in daily situations, people tend to skip or change phonemes when they pronounce the target word. Nonetheless, every talker speaks target words differently with a different tempo with different vocal apparatus, and listeners need to recognize the variety of speech sounds in order to understand what was being said (McQueen et al., 2006; Sjerps et al., 2011). In addition, the various forms of pronunciation can be also acoustically disrupted in daily situations. When it comes to second language learners’ speech perception, listeners need to know the vocabulary in the first place, followed by pronunciation variations and possible ambiguous speech sounds uttered by different talkers, and, lastly, listeners need to cope with any additional acoustic disruption in order to understand speech. While lexical context supports perceptual restoration, listeners need to know various pronunciation and possible acoustic disruption associated with the target words, and this is likely to be a challenging part of listening in the second language.

On the other hand, the current study also suggested that fricative-dominant items were significantly more intelligible than stop-dominant items in listener’s first language (L1 Japanese/L1 English), but not in listener’s second language (L2 English). To recall, fricative consonants have relatively symmetric waveforms as they are pronounced with the air constantly flowing out of the narrow constrictions in the vocal tract, and stop consonants have relatively asymmetric waveforms as they are pronounced with a noise burst in their articulation. So, when fricative-dominant lexical items were locally time-reversed, the amplitude envelope of speech relatively retained its original shape, and their intelligibility was also relatively preserved. On the other hand, when stop-dominant lexical items were locally time-reversed, the amplitude envelope of speech was more drastically disrupted, and their intelligibility was also more severely affected. It seems that the temporal envelope of speech signal contributes to the intelligibility of speech greatly (Ishida et al., 2016; Ishida et al., 2018; Stevens, 1999), but this fact seems to be enjoyed only when listeners are proficient in the target language (Kashino & Craig, 1994; Kashino, Van Wieringen, & Pols, 1992).

Ishida et al. (2018) also suggested, by using another method to modify the temporal envelope of speech, that the temporal envelope of speech is a key for perceptual restoration, and perceptual restoration is sustained by listeners’ language proficiency. In their study, the temporal envelope of speech was manipulated by low-pass filtering the modulation frequency components of speech signal that determine the shape of the amplitude envelope of speech (modulation-filtered speech). The low-pass cutoff frequency was 32, 16, 8, 4, 2, and 1 Hz, and the temporal envelope of speech signal lost its fine structure and configuration gradually when lower cutoff frequency was imposed. The biggest difference between locally time-reversed speech and modulation-filtered speech was that modulation-filtered speech retained the original temporal sequence of speech (i.e., temporal articulatory motion), although the modulation frequency components of speech signal were reduced. Their results suggested that native English speakers understood the modulation-filtered speech (spoken sentences) with 96, 95, 85, 44, 21, and 17% accuracy when speech signal was low-pass filtered at a cutoff frequency of 32, 16, 8, 4, 2, and 1 Hz respectively, while nonnative English speakers (lower intermediate proficiency) understood the modulation-filtered speech with 50, 44, 32, 19, 8, and 5% accuracy respectively. Here, there was a huge gap between native and nonnative speakers with 96% versus 50% accuracy even when the lightest disruption was imposed. It seems that the configuration of the temporal envelope of speech contributes to the intelligibility of speech, but listener’ proficiency is also a key—when listener’s proficiency is relatively high, the listeners should be able to use the spectrotemporal cues to perceptually restore temporally disrupted speech.

Additionally, the current study also suggested that perceptual restoration of locally time-reversed speech at a lexical level might be also affected by the language structure itself. The current study reported that the intelligibility of locally time-reversed fricative-dominant and stop-dominant stimuli in L1 Japanese was generally higher than that in L1 English reported in Ishida et al. (2016) (see Fig. 1). Both fricative-dominant Japanese words and pseudowords were intelligible even when the reversed segment length was 110 ms (*d'* = 2.49 for words and *d'* = 1.32 for pseudowords), and stop-dominant Japanese words were also intelligible even when the reversed segment length was 110 ms (*d'* = 1.64) while stop-dominant Japanese pseudowords was unintelligible (*d'* = −0.54). One possible reason why Japanese words and pseudowords were generally more tolerant of local time reversal and why listeners performed well in the same-different task, is that the Japanese language has CV as a basic linguistic unit, and this CV structure might have helped listeners to detect the subtle difference between words and pseudowords. The subtle difference between words and pseudowords in this study (i.e., only one consonant difference in a CVCVCVCV sequence in the second, third, or fourth consonant position) could have been detected relatively easily when the target consonant was surrounded by vowels. For example, when the target CV unit was composed of fricative + vowel, the phonological feature of these two phonemes is [+continuant], and the temporal envelope of speech signal (therefore, the intelligibility) would be relatively retained even when locally time-reversed. In this situation, it would be relatively easy to judge “same” and “different.” Also, when the target CV unit was comprised of stop + vowel, the phonological feature of these two sounds would be [−continuant] and [+continuant] respectively, and the temporal envelope of speech is more drastically changed. However, as the target stop consonant (second, third, or fourth consonant) was also surrounded by vowels in CVCVCVCV sequence in Japanese, it would be relatively easier for listeners to detect the difference in Japanese than in English. On the other hand, it should be also noted that “stop-dominant *pseudowords*” in L1 Japanese exhibited unintelligibility as “stop-dominant *pseudowords*” in L1 English did. It seems that stop-dominant stimuli with a CV structure can relatively retain their intelligibility even when locally time-reversed, but the intelligibility is lost if there is no lexical support. While previous studies of locally time-reversed speech at a sentence level did not exhibit the differences of intelligibility across different languages along with the extension of reversed segment length (when original speech was spoken at a moderate speech rate, and/or when speech rate was normalized; Greenberg & Arai, 2001; Ishida et al., 2018; Kiss et al., 2008; Remez et al., 2013; Stilp et al., 2010; Ueda et al., 2017), the current study of locally time-reversed speech at a lexical level (spoken at a moderate speech rate) reported different levels of intelligibility in L1 Japanese and L1 English. It is possible that the perceptual restoration at a lexical level can be affected by the language structure itself.

In fact, reanalysis of lexical items in this study suggested that the number of syllables or morae per word in English and Japanese was *not* statistically different, but the ratio of target phonemes per word was statistically different: The average number of syllables in fricative-dominant stimuli in English (*M* = 3.92, *SD* = 0.50) was not significantly different from the average number of morae in fricative-dominant stimuli in Japanese (*M* = 4.00, *SD* = 0.00), *t*(59) = −1.30, *p* = 0.20, 95% CI [−0.21, 0.05], *d* = 0.23. In addition, the average number of syllables in stop-dominant stimuli in English (*M* = 4.07, *SD* = 0.58) was not significantly different from the average number of morae in stop-dominant stimuli in Japanese (*M* = 4.00, *SD* = 0.00), *t*(59) = 0.89, *p* = 0.38, 95% CI [−0.08, 0.22], *d* = 0.17. However, the average ratio of fricatives per word in English (*M* = 20.19%, *SD* = 7.92) was significantly different from that in Japanese (*M* = 26.67%, *SD* = 4.29), *t*(90.81) = −5.57, *p* < .001, 95% CI [−8.79, −4.17], *d* = 1.02. Also, the average ratio of stops per word in English (*M* = 32.98%, *SD* = 6.01) was significantly different from that in Japanese (*M* = 40.63%, *SD* = 5.46), *t*(118) = −7.29, *p* < .001, 95% CI [−9.72, −5.57], *d* = 1.33. As the data show, the ratio of the target consonant occurrence in a word is different depending on the language, because different phonotactic rules govern each language. As English tends to include more phonemes in a word than Japanese, the ratio of target phonemes in a word seemed to be lower in English than in Japanese. This might also mean that the ratio of fricatives and stops per word also affected the intelligibility of locally time-reversed speech.

To recall, the ratio of fricatives and stops in words and pseudowords was higher in Japanese than in English, and the intelligibility of locally time-reversed lexical items in L1 Japanese was also generally higher than that in L1 English: The intelligibility of locally time-reversed “fricative-dominant *words*” in L1 Japanese, measured in *d'*, was 3.11, 2.96, 2.90, 2.75, 2.59, and 2.49 for the reversed segment length of 10, 30, 50, 70, 90, and 110 ms, respectively, while that in L1 English was 2.90, 2.81, 2.13, 1.88, 1.02, and 0.97, respectively. Also, the intelligibility of locally time-reversed “fricative-dominant *pseudowords*” in L1 Japanese, measured in *d'*, was 2.65, 2.29, 1.96, 1.81, 1.67, and 1.32 for the reversed segment length of 10, 30, 50, 70, 90, and 110 ms, respectively, while that in L1 English was 2.26, 1.46, 0.79, 0.46, 0.30, and 0.17, respectively. Here, the higher intelligibility of locally time-reversed fricative-dominant words and pseudowords in L1 Japanese might reflect the higher ratio of fricatives per word in Japanese than in English. In addition, the intelligibility of locally time-reversed “stop-dominant *words*” in L1 Japanese, measured in *d'*, was 2.77, 2.42, 2.41, 1.99, 1.85, and 1.64 for the reversed segment length of 10, 30, 50, 70, 90, and 110 ms, respectively, while that in L1 English was 3.08, 2.72, 2.38, 1.77, 1.40, and 0.86, respectively. Here, the intelligibility of locally time-reversed stop-dominant words was generally higher in L1 Japanese, and this might also reflect the CVCVCVCV structure of the Japanese stimuli where a consonant is usually surrounded by vowels, and, therefore, the temporal envelope of speech as well as the intelligibility of speech is relatively retained even after the local time reversal. At the same time, the intelligibility of locally time-reversed “stop-dominant *pseudowords*” in L1 Japanese, measured in *d'*, was 1.47, 0.49, −0.70, −0.74, −0.85, and −0.54, for the reversed segment length of 10, 30, 50, 70, 90, and 110 ms, respectively, while that in L1 English was 2.40, 0.84, −0.15, 0.13, −0.22, and −0.27, respectively. Here, both L1 Japanese and L1 English exhibited the unintelligibility of locally time-reversed “stop-dominant *pseudowords*” when the reversed segment length exceeded 30 ms. When participants were confronted by locally time-reversed stop-dominant pseudowords where there is no lexical context, listeners seem to be biased to respond “same” or “different” in a wrong way in the same-different task. The composition of the stimuli, which also reflects linguistic differences between the Japanese and English languages, seems to largely affect the intelligibility of locally time-reversed lexical items.

As a matter of fact, the basic structure of English and Japanese is greatly different. For example, in English, a syllable is a basic linguistic unit, and a syllable has various structures such as V, VC, VCC, VCCC, CV, CVC, CVCC, CVCCC, CVCCCC, CCV, CCVC, CCVCC, CCVCCC, CCCV, CCCVC, CCCVCC, and CCCVCCC (see Table 1; Bergman, Hall, & Ross, 2007; Celce-Murcia, Brinton, & Goodwin, 2010; Kono, 2004). On the other hand, in Japanese, a mora is a basic linguistic unit, and it has only a few basic structures such as V and CV, while C and CCV are possible in some special contexts (see Table 1; Kawagoe, 2007; Kono, 2004). Therefore, the number of target consonants appearing in each word (fricatives or stops) can differ, and the ratio of these target phonemes can also differ. These differences possibly affect the intelligibility of locally time-reversed speech.

**Table 1:**
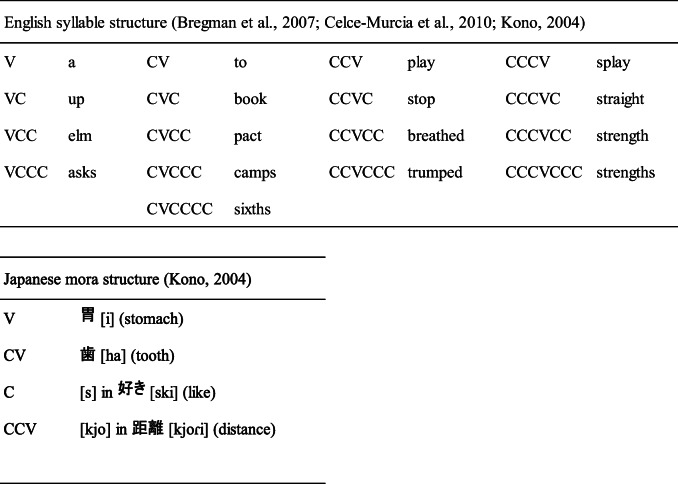
Comparison between the English syllable structure and the Japanese mora structure

Furthermore, perceptual restoration of locally time-reversed lexical items can be also affected by a sound unit in a language. For example, when it comes to a proper noun such as “London,” native English speakers would pronounce this word as /l∧ndən/ with two syllables, but native Japanese speakers would pronounce this word in Japanese (“ロンドン”) as /roNdoN/ with four morae such as /ro/, /N/, /do/, and /N/ (Kawagoe, 2007). Also, native English speakers would pronounce “Starbucks” as /stɑrb∧ks/ with two syllables and “coffee” // with two syllables, while native Japanese speakers would pronounce these words in Japanese (“スタ―バックス” and “コ―ヒ―”) as /sɯtaabaQkɯsɯ/ with seven morae and /kooçii/ with four morae respectively. Therefore, it is possible that the basic temporal unit listeners would feel and/or are familiar with is different in each language, and the perceptual tolerance to local time reversal is also related to the basic temporal unit in each language. When listeners need to deal with two languages that have different temporal units as well as linguistic structures, that probably means listeners need to adjust their auditory perception and perceptual restoration depending on the language, and this can be also a challenging process for L2 listeners (i.e., intermediate level L2 listeners in this study).

Put another way, the way people perceptually restore the disrupted portion of speech can be largely influenced by listeners’ native language. Kashino (1990) examined the perceptual restoration of intervocalic stop consonant of /p/, /t/, and /k/ by using a pseudoword VCV sequence (e.g., /ate/) uttered by native Japanese speakers. In his study, native Japanese listeners heard a VC sequence (preclosure portion) and a CV sequence (postclosure portion) extracted from the VCV sequence. For VC stimuli, the preclosure portion was replaced by either 10, 30, 50, or 70-ms noise from the closure point of the stop consonant. For CV stimuli, the postclosure portion was replaced by either 10, 30, 50, or 70-ms noise from the burst release of the stop consonant. The results suggested that listeners had a great difficulty identifying the consonant in a VC sequence, and, even without noise, listeners could identify the consonant with only 67% accuracy. On the other hand, listeners relatively easily identified the consonant in a CV sequence, and, without noise, listeners could identify the consonant with 91% accuracy. As the duration of replacing noise increased, the identification rate dropped progressively in both VC and CV conditions, but the identification rate in the CV condition was always higher than that of the VC condition. It seems to suggest that native Japanese listeners tend to use postclosure information when perceptually restoring and/or identifying the intervocalic consonant, and this is because the Japanese language has a CV as a basic linguistic unit.

Kashino (1990) also examined the perceptual restoration of intervocalic stop consonant /p/, /t/, and /k/ in other conditions: (1) “VCV” condition where the pseudoword sequence VCV was articulated by a native Japanese speaker, (2) “V + CV” condition in which a postclosure sequence of CV was extracted from a VCV sequence and combined with a Japanese vowel pronounced as a single sound, and (3) “VC_1_C_2_V” condition in which VC_1_ (preclosure portion) from a VC_1_V sequence (e.g., /ate/) and C_2_V (postclosure portion) from a VC_2_V (e.g., /ape/) were combined together. In these conditions, the postclosure portion of CV was replaced by 10, 30, 50, or 70-ms noise. The results suggested that, in the VCV condition (where both preclosure and postclosure information was originally available), listeners identified the intervocalic stop consonant relatively easily, with above 80% accuracy even when the postclosure portion was replaced by 70-ms noise. On the other hand, in the “V + CV” condition (where no preclosure information was available), listeners identified the target consonants with lower accuracy than the VCV condition but higher accuracy than the CV condition. It seems that the presence of a vowel supported the perception of the intervocalic stop consonant to some extent even when the vowel sound did not contain preclosure information. When it comes to the perception of the intervocalic consonant in the VC_1_C_2_V condition, native Japanese listeners perceived C_2_ when there was no noise. In addition, C_2_ was still perceived more than C_1_ when the postclosure portion of C_2_ was replaced by 10-ms and 30-ms noise. The perception rate for C_1_ and C_2_ was almost the same when the postclosure portion of C_2_ was replaced by 50-ms noise, and, finally, the perception rate for C_1_ was higher than that for C_2_ when the postclosure portion was replaced by 70-ms noise. Overall, native Japanese listeners tend to perceive C_2_ more in VC_1_C_2_V sequence, and it seems to imply that native Japanese listeners tend to use the perceptual cues originating from a CV sequence that should be familiar to native Japanese listeners as CV is the basic linguistic unit in Japanese.

Kashino et al. (1992) further examined the perceptual restoration of the Japanese intervocalic stop consonant /p/, /t/, and /k/ by native Japanese and Dutch listeners. This study adopted the stimuli of Kashino (1990), and examined how Japanese and Dutch listeners perceive the Japanese intervocalic stop consonant in a VCV, CV, VC, and VC_1_C_2_V sequence where the postclosure portion was replaced by 10, 30, 50, or 70-ms noise. The results suggested that both Japanese and Dutch listeners perceived the intervocalic stop consonant better in the VCV condition than in the CV condition, suggesting that both Japanese and Dutch listeners can use preclosure information when perceptually restoring the intervocalic stop consonant. On the other hand, Dutch listeners identified the intervocalic stop consonant in the VC and VC_1_C_2_V better than Japanese listeners, which suggests that Dutch listeners might be more adept at utilizing the VC cues in perception. Put another way, Japanese listeners might have performed worse than Dutch listeners because the Japanese language is an open-vowel language and does not have VC as a linguistic unit. While the perceptual cues are distributed before and after the target speech sounds as the articulatory gestures are continuous, it is possible that listeners’ perception is largely regulated by the linguistic structure of their first language.

Grataloup et al. (2009) also suggested that perceptual restoration of locally time-reversed speech seems to be regulated by basic linguistic units. In their study, perceptual restoration of locally time-reversed words and pseudowords were examined by using disyllabic French words (common nouns) and pseudowords (that contained identical syllables that appeared in words). The disyllabic words and pseudowords had a structure of CV–CV, CV–CCV, and CCV–CV, and the boundary of syllables was identified by a native French speaker, and the temporal midpoint of a syllable was also defined as a half syllable. In their study, French listeners listened to words and pseudowords in which the local time reversal was imposed at a 0.5, 1, 1.5, and 2 syllables. In other words, there was a natural temporal sequence of speech remained in the stimuli if only 0.5, 1, and 1.5 syllables were reversed. Their results suggested that the average intelligibility was 97.3%, 79.7%, 51.2%, 2.9%, and 1.8% when 0, 0.5, 1, 1.5, and 2 syllables were reversed, respectively. Here, the intelligibility of locally timer-reversed words and pseudowords dropped by half when the first syllable was reversed and the second syllable remained the same. In addition, words and pseudowords became unintelligible when 1.5 syllable were reversed and only 0.5 syllable remained the same. It seems that the intelligibility of speech drastically dropped when the temporal reversal was imposed beyond a syllable boundary. By using a syllable as a unit of temporal distortion, this study suggested that a syllable contributes to the intelligibility of speech, and our perception system might be also regulated by a temporal unit that each language has, which is conventionally discussed in the domain of language rhythms such as stress-timed language, syllable-timed language, and mora-timed language, although the classification of language rhythms is debatable (Arvaniti, 2012; Loukina, Kochanski, Rosner, Keane, & Shih, 2011; Otake, 2015; Polyanskaya & Ordin, 2015). Additionally, this study also suggested the lexical effects in perceptual restoration—words were perceptually restored better than pseudowords. Again, lexicality largely influences the intelligibility of speech, while the basic temporal unit in each language (which can be linguistically discussed as a phoneme, syllable, mora, etc.) also seems to affect perceptual restoration processes. It is possible that top-down contextual cues and bottom-up acoustic cues are combined in perceptual restoration processes, but differently by native and nonnative listeners.

Lastly, Voss (1984) argued, with his study of *slips of the ear* where speech was misunderstood (or inaccurately perceptually restored), that native speakers had advantages when perceptually restoring the unclear portion of speech as they could process “acoustic,” “linguistic,” and “content” components of the language better than nonnative speakers. Listeners can have a good command of “bottom-up,” “top-down” and “heterarchical” processing of speech in their first language when interpreting incoming speech signal. Also, Bond (1999) suggested, with her studies of *slips of the ear*, that listeners utilize their “knowledge about the language, the speaker, and the conversation” (p.127) when perceptually restoring the unclear portion of speech. Listeners integrate acoustic, linguistic, and contextual information when understanding the ambiguous parts of speech. When listeners need to perceptually restore the drastic acoustic distortion of speech as locally time-reversed speech, they need to be proficient in the target language.

In summary, the current study suggested that the same individuals (native Japanese speakers who learned English as a second language) perceptually restored locally time-reversed words and pseudowords better in their first language (L1 Japanese) than in their second language (L2 English). In addition, words were more tolerant of local time reversal than pseudowords in both listeners’ first and second language. On the other hand, acoustic-phonetic properties (fricative vs. stop) affected the intelligibility of locally time-reversed speech significantly in listeners’ first language but not significantly in listeners’ second language. The perceptual restoration seems to be largely regulated by lexical contexts, while acoustic-phonetic properties are coming into play when listeners are familiar with the target language, and when listeners are able to direct their attention to the acoustic details of speech. Lastly, it is interesting to note that the intelligibility of locally time-reversed words and pseudowords in L1 Japanese was relatively higher than that in L1 English reported in Ishida et al. (2018). In the future study, it might be interesting to examine how native English speakers who learn Japanese as a second language would perceptually restore locally time-reversed words and pseudowords in their L2 Japanese in order to compare their performance in L2 Japanese with the current results in L2 English. For now, the current study tentatively concludes that different languages can exhibit different levels of intelligibility when confronted with local time reversal at a lexical level, and this intelligibility gap at a lexical level seems to be arising from the differences in language structure and listeners’ language proficiency.

## Acknowledgements

I would like to thank Dr. Arty Samuel and Dr. Takayuki Arai for their insightful comments and support with this project. I also would like to thank all the participants for this project. This study was supported by Grant-in-Aid for JSPS Fellows # 15J00033 & 17J00285 and Keio University Academic Development Funds for Individual Research.

## Data Availability

Materials created and used for this study were described in the manuscript along with appendices. The data obtained during this study were reported in the manuscript along with figures and tables. Any additional information, materials, and datasets are available from the corresponding author.

## Appendix

**Table 2:** Words and pseudowords from Ishida et al. (2016)

Stop-dominant words	Stop-dominant pseudowords	Fricative-dominant words	Fricative-dominant pseudowords
academic	aca**b**emic	additional	a**b**itional
acceptable	ac**sh**eptable	artificial	ar**p**ificial
activity	ac**p**ivity	associate	a**sh**ociate
anticipate	an**p**icipate	authority	authori**p**y
appointment	a**t**ointment	available	availa**d**le
architecture	archi**p**ecture	avenue	ave**m**ue
attitude	atti**p**ude	citizen	ci**p**izen
capable	ca**t**able	civilization	civili**zh**ation
capacity	ca**t**acity	confirmation	confir**n**ation
category	ca**p**egory	conventional	conven**s**onal
comfortable	comfor**p**able	conversation	conver**sh**ation
commodity	co**n**odity	definition	defi**m**ition
companionship	com**t**anionship	easily	ea**zh**ily
company	com**t**any	education	edu**t**ation
competition	compe**p**ition	emotional	e**n**otional
considerable	consi**b**erable	environment	environ**n**ent
contemporary	con**p**emporary	evolution	evolu**s**on
decorated	de**t**orated	experience	e**ksh**perience
deliberate	deli**d**erate	foundation	foun**b**ation
department	de**t**artment	generosity	ge**m**erosity
development	develop**n**ent	information	infor**n**ation
difficulty	diffi**t**ulty	inheritance	inheri**p**ance
disappointment	disa**t**ointment	insurance	in**ss**urance
documentary	docu**n**entary	intelligence	in**p**elligence
educated	edu**t**ated	international	inter**m**ational
electricity	electri**sh**ity	intervention	in**p**ervention
entertainment	enter**p**ainment	invitation	invi**p**ation
executive	e**gzh**ecutive	isolation	i**sh**olation
experiment	ex**t**eriment	machinery	machi**m**ery
extraordinary	extraordi**m**ary	magnificent	mag**m**ificent
identical	i**b**entical	mechanism	mecha**m**ism
important	im**t**ortant	miserable	mi**zh**erable
incapable	in**t**apable	misery	mi**zh**ery
incredible	incre**b**ible	monastery	mo**m**astery
independence	inde**t**endence	mysterious	my**sh**terious
interpretation	interpre**p**ation	national	na**s**onal
interrupted	in**p**errupted	necessary	nece**sh**ary
introduction	intro**b**uction	occasional	o**t**asional
nobody	no**d**ody	officer	offi**sh**er
opportunity	oppor**p**unity	official	offi**s**al
particular	par**p**icular	operation	o**t**eration
political	poli**p**ical	opposition	o**t**osition
possibility	possi**d**ility	organization	orga**m**ization
predicament	pre**b**icament	personally	per**sh**onally
profitable	profi**p**able	philosophy	philo**sh**ophy
propaganda	propa**d**anda	professional	profe**s**onal
property	pro**t**erty	psychology	psy**t**ology
publication	publi**t**ation	radiation	ra**b**iation
publicity	publi**sh**ity	reasonable	reaso**m**able
remarkable	remar**t**able	reception	re**sh**eption
representative	represen**p**ative	relationship	rela**s**onship
republican	re**t**ublican	revolution	revolu**s**on
respectable	res**t**ectable	scientific	scien**p**ific
sophisticated	sophisti**t**ated	separation	se**t**aration
spectacular	spec**p**acular	society	so**sh**iety
stability	sta**d**ility	superficial	su**t**erficial
understandable	understan**b**able	supervisor	su**t**ervisor
understatement	understate**n**ent	universal	u**m**iversal
unexpected	unex**t**ected	university	u**m**iversity
unpredictable	unpre**b**ictable	whatsoever	what**sh**oever

**Table 3:** Words from Amano and Kondo (2000) and their matched pseudowords

Stop-dominant words	Stop-dominant pseudowords	Fricative-dominant words	Fricative-dominant pseudowords
bakuyaku	ba**p**uyaku	hishigata	hi**h**igata
butaniku	bu**p**aniku	hikidashi	hikida**h**i
datemaki	da**p**emaki	hitosama	hito**h**ama
dekasegi	de**p**asegi	hitohada	hito**s**ada
dekigoto	de**p**igoto	hiyakashi	hiyaka**h**i
dekitate	deki**p**ate	hirusugi	hiru**f**ugi
dokudami	doku**b**ami	hirumeshi	hirume**h**i
dokugaku	doku**d**aku	hahakata	ha**s**akata
dokuhebi	do**p**uhebi	hahanohi	ha**s**anohi
gakubuchi	ga**p**ubuchi	hakanasa	hakana**h**a
gakureki	ga**p**ureki	hagishiri	hagi**h**iri
garakuta	gara**p**uta	hakidasu	hakida**f**u
gekitotsu	ge**p**itotsu	hakuhatsu	haku**s**atsu
getsugaku	getsu**d**aku	hageshisa	hage**h**isa
gobusata	go**g**usata	hachinosu	hachino**f**u
gokubuto	go**p**ubuto	hanashite	hana**h**ite
gokuraku	go**p**uraku	hanafuda	hana**s**uda
kabekake	kabe**p**ake	harusaki	haru**h**aki
kakesoba	ka**p**esoba	harusame	haru**h**ame
kakutoku	ka**p**utoku	hesokuri	he**h**okuri
kamaboko	kamabo**t**o	hoshigaki	ho**h**igaki
kamigata	kami**d**ata	hoshigaru	ho**h**igaru
katagaki	ka**p**agaki	hoshikusa	ho**h**ikusa
katakana	kata**p**ana	hosomeru	ho**h**omeru
katakoto	kata**p**oto	horidasu	horida**f**u
kigakari	kiga**p**ari	horobosu	horobo**f**u
kigokochi	kigo**p**ochi	fushimatsu	fu**h**imatsu
kikikata	ki**p**ikata	fukashigi	fuka**h**igi
kikitori	kiki**p**ori	fukisoku	fuki**h**oku
kikubari	kiku**d**ari	fukidasu	fukida**f**u
kirikabu	kiri**p**abu	fuyufuku	fuyu**s**uku
kobutori	ko**g**utori	furisode	furi**h**ode
kodakara	ko**g**akara	furidashi	furida**h**i
kokuhaku	ko**p**uhaku	furusato	furu**h**ato
kokuseki	ko**p**useki	furoshiki	furo**h**iki
kotobuki	koto**g**uki	sasageru	sa**h**ageru
kotogara	ko**p**ogara	sashihiki	sashi**sh**iki
kuchibeta	kuchi**g**eta	sakaseru	saka**h**eru
kugiduke	ku**b**iduke	sakihodo	saki**s**odo
kujibiki	kuji**g**iki	sakeguse	sakegu**h**e
kumitate	kumi**p**ate	sabishige	sabi**h**ige
kitaguni	ki**p**aguni	seseragi	se**h**eragi
kuzukago	kuzu**p**ago	sekisetsu	seki**h**etsu
tabibito	tabi**g**ito	setsunasa	setsuna**h**a
takatobi	ta**p**atobi	shizukesa	shizuke**h**a
takenokoto	ta**p**enokoto	shitashisa	shita**h**isa
takoyaki	ta**p**oyaki	shitsukosa	shitsuko**h**a
tanabata	tana**g**ata	shibutosa	shibuto**h**a
tategaki	tate**b**aki	shimedasu	shimeda**f**u
tebukuro	te**g**ukuro	shiraseru	shira**h**eru
tedasuke	te**b**asuke	shiromiso	shiromi**h**o
tekagami	teka**b**ami	shiwayose	shiwayo**h**e
tekikaku	teki**p**aku	susumeru	su**f**umeru
tetsugaku	tetsu**d**aku	suzushige	suzu**h**ige
tobibako	tobi**d**ako	suzumushi	suzumu**h**i
tobikomi	to**g**ikomi	sunahama	suna**s**ama
tokubetsu	toku**g**etsu	subayasa	subaya**h**a
tokuyaku	to**p**uyaku	surudosa	surudo**h**a
tonogata	tono**b**ata	sokonashi	sokona**h**i
toritate	tori**p**ate	sobakasu	sobaka**f**u
